# WWP1 E3 ligase at the crossroads of health and disease

**DOI:** 10.1038/s41419-023-06380-0

**Published:** 2023-12-21

**Authors:** Abhayananda Behera, Aramati Bindu Madhava Reddy

**Affiliations:** Department of Animal Biology, School of Life Sciences, University of Hyderabad, Hyderabad, 500046 India

**Keywords:** Ubiquitylation, Cancer metabolism

## Abstract

The E3 ubiquitin ligase WWP1 (WW Domain-containing E3 Ubiquitin Protein Ligase 1) is a member of the HECT (Homologous to the E6-associated protein Carboxyl Terminus) E3 ligase family. It is conserved across several species and plays crucial roles in various physiological processes, including development, cell growth and proliferation, apoptosis, and differentiation. It exerts its functions through ubiquitination or protein-protein interaction with PPXY-containing proteins. WWP1 plays a role in several human diseases, including cardiac conditions, neurodevelopmental, age-associated osteogenic disorders, infectious diseases, and cancers. In solid tumors, WWP1 plays a dual role as both an oncogene and a tumor suppressor, whereas in hematological malignancies such as AML, it is identified as a dedicated oncogene. Importantly, WWP1 inhibition using small molecule inhibitors such as Indole-3-Carbinol (I3C) and Bortezomib or siRNAs leads to significant suppression of cancer growth and healing of bone fractures, suggesting that WWP1 might serve as a potential therapeutic target for several diseases. In this review, we discuss the evolutionary perspective, structure, and functions of WWP1 and its multilevel regulation by various regulators. We also examine its emerging roles in cancer progression and its therapeutic potential. Finally, we highlight WWP1’s role in normal physiology, contribution to pathological conditions, and therapeutic potential for cancer and other diseases.

## Facts

- WWP1 is a HECT E3 ubiquitin ligase that targets multiple substrates for ubiquitination.
- Most of the WWP1 interacting partners (substrates) have PY motifs (e.g., RUNX2, KLF5, P63, etc.).
- WWP1 plays a pivotal role in diverse normal physiological processes and is implicated in various diseases, including neurological disorders, infectious diseases, and cancer.
- In cancer, WWP1 often exhibits overexpression or hyperactivation, a trend associated with unfavorable patient prognoses. Preclinical data suggest that WWP1 could be a potential therapeutic target for cancer and various other diseases.

## Open questions

- How does WWP1 govern normal physiological processes? Is the physiological function of WWP1 evolutionarily conserved across different species?
- How does WWP1 influence the development of several diseases and cancer?
- What are the underlying mechanisms responsible for the overexpression, hyperactivation, or autoinhibition of WWP1 in cancer?
- How can WWP1 be strategically targeted to effectively mitigate disease burden and improve patient survival? Can the clinical translation of WWP1 modulation be realized?

## Evolutionary history of WWP1

In 1994, Marius Sudol and his group identified a novel 38 aa long domain within the YAP (Yes-associated protein) oncogene. This domain was named the ‘WW domain’ due to the presence of two tryptophan (W) residues within its structure [1, 2]. It is expressed in several signaling and regulatory molecules, including dystrophin, utrophin, and NEDD-4 (Neural precursor cells expressed developmentally downregulated), and is conserved among various species [2]. In 1997, Pirozzi et al. discovered three distinct WWPs, namely WWP1, WWP2, and WWP3, using a method called cloning of ligand targets (COLT) from the human brain and bone marrow [3]. They found that WWP1 and WWP2 contain C-terminal HECT domains with E3 ubiquitin ligase activity. In 1998, Wood et al. discovered five interactomes of atrophin-1, a protein responsible for a neurodegenerative disorder known as DRPLA (Dentatorubral and pallidoluysian atrophy) [4]. All five atrophin-1 interacting proteins (AIPs) express the WW domain, which interacts with the PY motifs (PPXY) of atrophin-1. Furthermore, three of these AIPs, namely WWP1 (AIP5), WWP2 (AIP2), and WWP3 (AIP3), express class I WW domains [5]. Unlike WWP3; WWP1 and WWP2 are well-studied HECT E3 ligases. Both WWP1 and 2 have four tryptophan (WW) domains that interact with PPXY-containing target substrates [1, 6]. They can also interact with common PY motif-containing substrates involved in viral budding through ubiquitination of vacuolar protein sorting factors [7, 8] as well as with atrophin-1 and PTEN. Hence, they are referred to as AIP5 (WWP1) and AIP2 (WWP2) [4]. Unlike WWP1 and WWP2, WWP3 features two WW domains only and lacks E3 ubiquitin ligase activity. Additionally, its guanylate kinase domain is functionally inactive [9]. WWP3/MAGI1 is involved in integrin-mediated cell-cell adhesion, acting as a scaffold between tight junctions and adherens junctions in both epithelial and endothelial cells [9, 10].

**Fig. 1:**
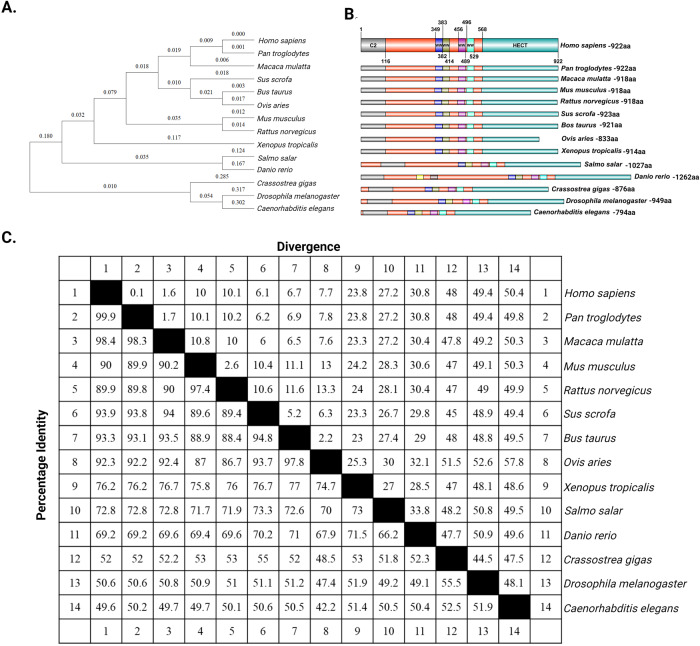
*WWP1* is an evolutionarily well-conserved gene. **A**. Evolutionary relationships of WWP1 across various species. The amino acid sequence of WWP1 is aligned using the ClustalW algorithm, and the evolutionary history was inferred using the Neighbor joining method. The tree is drawn to scale, with branch lengths in the same units as those of the evolutionary distances used to infer the phylogenetic tree. The evolutionary distances were computed using the Dayhoff matrix-based method and are in the units of the number of amino acid substitutions per site. All positions containing alignment gaps and missing data were eliminated only in pairwise sequence comparisons (pairwise deletion option). Phylogenetic analyses were conducted in MEGA11. **B** The schematic representation of the structure of WWP1 was shown using the InterPro Scan (https://www.ebi.ac.uk/interpro/about/interproscan) and DOG 2.0 (http://dog.biocuckoo.org/) databases. The C2 domain, four WW domain and HECT domain are conserved from lower organisms to higher organisms were shown. **C** Percentage identity of evolutionarily conserved WWP1 from different species.

Evolutionary biology offers valuable insights into the origin of the gene, conservation and comparison of protein sequences among diverse species; reveals critical conserved regions, species-domain-substrate-specific functional significance [11–13]. *WWP1* has variable gene and intron lengths but a relatively conserved exon structure and functional domains. A phylogenetic tree of variants of WWP1 found in different species, constructed using multiple sequence alignment tools (Fig. 1A, B) was used to evaluate the similarity and divergence among different species (Fig. 1C). The analysis showed that WWP1 is more than 90% conserved in mammals where as 50% less conserved in lower organisms. Interestingly, WW domains and the catalytic HECT domain exhibit a high degree of conservation, spanning from lowers animal like worms to higher vertebrates such as mammals [14]. Exceptions include *Ovis aries*, which has a smaller and less conserved HECT, and *Danio rerio*, which has six WW domains (Fig. 1B). Marin I’s studies on the evolution of animal HECT ubiquitin ligases have revealed that large HERCs and small HERCs are evolutionarily very distant despite their structural similarities, which are due to a convergence phenomenon. The diversification of HECT E3 in animals occurred just before or at the time of the choanoflagellate/metazoan split. This diversification, achieved through the loss or duplication of substrate-specific domains, allowed these enzymes to either regulate newly evolved signaling pathways during animal evolution or adapt to existing ones as their substrates [14]. To understand the function of these genes across evolution, one can examine changes in gene length, the presence of multiple domains within a single gene, and the repetition of specific domains. However, determining the precise significance of gene function in each species would require specific experimental systems.

The literature shows that WWP1 has functional similarity across various species. Huang et al. investigated the physiological function of murine (*mWwp1*) and *Caenorhabditis elegans* (*C. elegans*) (*CeWwp1*) Wwp1 clones ortholog of *hWwp1*. The authors revealed that RNAi-mediated silencing of *CeWwp1* in *C. elegans* resulted in significant morphological defects and late embryonic lethality, suggesting that CeWWP1 is essential for morphogenesis during embryogenesis in *C. elegans* [15]. WWP1 plays crucial roles in diverse physiological processes such as patterning of vulval precursor cells in *C. elegans* [16], bone development [17–22], neuron development [23, 24] and epithelial sodium ion channels in humans [25, 26]. Additionally, WWP1 is involved in virus internalization [27], and budding [8, 28–32]. Recent studies have indicated that WWP1 interacts with the PPXY motif (25-PPAY-28) of the SARS-CoV-2 spike protein, leading to its ubiquitination and regulation of SARS-CoV-2 function. Additionally, germline variants of WWP1 are significantly associated with severe cases of COVID-19 [33]. WWP1 is also implicated in the dysregulation of solid tumors and hematological malignancies, impacting patient survival [34, 35]. Indole-3-Carbinol (I3C), derived from cruciferous vegetables (*Brassicaceae* family), can inhibit WWP1 enzymatic activity, making it a promising candidate for antiviral and anticancer treatments [33, 36]. In summary, the intricate interplay between WWP1, its role in cancer and viral-related processes, and the potential therapeutic implications of compounds like I3C highlight the multifaceted scope of its biological roles and the promise it could hold for future therapeutic development.

## Ubiquitination process and HECT E3 ubiquitin ligases

Ubiquitination is a major posttranslational modification of protein substrates that involves the covalent attachment of ubiquitin (Ub) to a target protein. Depending on the number of Ub molecules attached to the substrate, ubiquitination is generally categorized into three types - monoubiquitination (addition of one Ub to a single residue of the substrate), multi-monoubiquitination (addition of several Ub molecules to different sites of the substrate), and polyubiquitination (addition of several Ub molecules to a single site of the substrate) [37]. Polyubiquitination can be either linear or branched. Ubiquitin is a small regulatory protein composed of 76 amino acids, containing seven lysine residues (K6, K11, K27, K29, K33, K48 and K63). Among these lysine residues, one plays a pivotal role in Ub-Ub conjugation within polyubiquitylated proteins, resulting in seven homotypic Ub linkages and multiple heterotypic Ub linkages. These diverse ubiquitin linkages determine the fate of proteins, including aspects such as protein abundance, cellular localization, protein-protein interaction, among others. For example, proteins conjugated with lysine-11 or lysine-48 are mainly targeted for protein degradation, whereas proteins tagged with lysine-63 are stabilized and participate in various cellular signaling pathways [37].

The ubiquitination process follows a three-step enzymatic cascade. First, E1 (Ub-activating enzyme) activates Ub. Second, the Ub-conjugating enzyme E2 transfers the activated Ub from E1 to the active site of E2. Finally, the Ub-ligase E3 transfers the Ub to the target protein. E3 ligases are critical components of ubiquitination and mediate substrate specificity. E3 ubiquitin ligases are classified into three categories based on their structure and ubiquitin transfer mode: (1) HECT, (2) RING (Really interesting new gene), and (3) RBR (RING-in-between-RING) E3 ligases [38]. Among the 700 ubiquitin E3 ligases encoded in the mammalian genome, there are 28 HECT type E3 ligases. HECT E3 ligases can be further divided into 3 types based on their N-terminal structure, namely NEDD4, HERC (HECT and RCC1-like domain), and other HECT ligases. The most studied HECT E3s are the NEDD4 subfamily, which consists of nine members: NEDD4-1, NEDD4-2, WWP1, WWP2, ITCH, NEDL1, NEDL2, SMURF1, and SMURF2 [34, 39]. Their N-terminal has a C2 domain for membrane binding, 2–4 WW domains for the recognition of PPXY-containing substrates, and a C-terminal HECT domain for the transfer of Ub to substrates. NEDD4.1, NEDD4.2, ITCH, WWP1 and WWP2 have four WW domains. SMURF2 has three WW domains, whereas SMURF1, NEDL1 and NEDL2 have two WW domains [7]. Understanding the diversity of E3 ubiquitin ligases is essential for comprehending the complexities of protein regulation within the cell.

## Structure and activation of WWP1

**Fig. 2:**
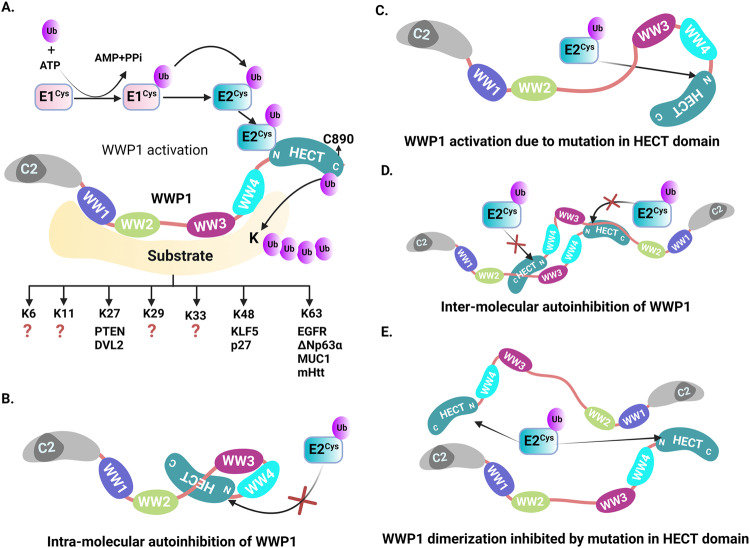
Activation and autoinhibition of WWP1. **A** The cartoon depicts WWP1 E3 ligase transfers ubiquitin (Ub) molecules from E2 to E3, finally from E3 to its target substrates. Ub binds to catalytically active cysteine site of the E1 activating enzyme in an ATP-dependent manner. The E1 enzyme transfers the ubiquitin molecule to the catalytically active cysteine site of the E2 conjugating enzyme. E2 transfers the Ub molecule to the catalytically active cysteine site of E3 ubiquitin ligating enzyme by interacting with the N-terminus site of the WWP1 HECT domain. Finally, HECT domain transfers the Ub to the targeting substrate, which interacts with the WW domain of the WWP1. WWP1 directs different polyubiquitination linkages of its substrates: K27 polyubiquitination of PTEN and DVL2, K48 linkage of p27 and KLF5, K63-linked polyubiquitination of EGFR MUC1 etc. However, whether WWP1 directs polyubiquitination of its substrates through other linkages (K6, K11, K29 and K33) remains to be identified (denoted by a red question mark). **B** Wild-type WWP1 is autoinhibited through intramolecular interaction by sequestering its HECT domain in between the 2,3-linker and WW2-WW3 domains. **C** Mutations in the HECT domain disrupt the intramolecular autoinhibitory activity of WWP1 leading to its activation. **D** Intermolecular autoinhibition is carried out by the HECT domain of WWP1 which is sequestered in between the WW2 to WW3 linker domain. **E** The mutation in both the HECT domain inhibits the dimerization of WWP1.

WWP1 is encoded by the gene *WWP1*, which is located in the long arm of chromosome 8 (8q21.3), adjacent to the proto-oncogene *MYC* in humans, and consists of 26 exons [25]. The size of the full-length mRNA is around 4.2 kb, while the open reading frame is 2766 bp. (https://www.ncbi.nlm.nih.gov/nuccore/NM_007013.4) [40, 41]. Alternative splicing of *WWP1* generates at least six isoforms with or without an N- terminal C2 domain by removing its predicted C-terminal β strands [42]. The predominant isoform of WWP1 is 922 amino acids long (predicted molecular weight is 110 kDa) [43]. WWP1 is highly conserved among various species from humans to *C. elegans* (Fig. 1). WWP1 has an N-terminal C2 domain, followed by four middle WW domains (WW 1–4), and a C-terminal HECT domain (Fig. 2A). The C2- domain interacts with the lipid molecules in a Ca2+-dependent manner and is responsible for membrane targeting.[44, 45]. Each WW domain contains 38–40 semi-conserved amino acid residues with two W (tryptophan) residues (20–22 residues apart) in the form of a triple-strand β-sheet [1, 46]. These domains engage in protein-protein interactions through their aromatic amino acid residues [47–49], thus serving as scaffolds for recruitment and subcellular localization of target proteins [50]. WWP1 utilizes its four WW domains to interact with substrates containing PPXY (phospho-Ser-Pro and Pro-Arg) motifs, marking them with ubiquitin. It can also interact with substrates lacking PY motifs, which require adapter proteins for ubiquitination [51]. These substrates encompass TβR1, SMAD4, KLF2, Shn3, p53, p27, TGIF, and EPS15 [52]. During ubiquitination, several SMAD proteins can serve as adapters for WWP1’s E3 ubiquitin ligase activity. For instance, WWP1 induces RUNX2 degradation via SMAD6 [53] and SMAD2 can function as an adapter protein for WWP1 to induce SMAD4 ubiquitination [54, 55]. WWP1 also recruits other E3 ubiquitin ligases for the ubiquitination of KLF2 [56], while Shn3 can act as an adapter for RUNX2 degradation by recruiting WWP1 [17]. WW domains- 1 and - 3, are type-I WW domains that interact strongly with PY motifs, whereas WW domains- 2 and - 4 show weaker binding to these motifs [20, 49, 57, 58]. The C-terminal HECT domain in WWP1 consists of two lobes – N and C- lobes. It possesses E3- ligase activity and binds to ubiquitin-conjugating enzymes. The ligase activity of WWP1, responsible for ubiquitinating target proteins, depends on the rotation of the C-lobe around a polypeptide hinge that connects it to the C- lobe [58, 59]. Unlike RING E3 ligases, WWP1 catalyses ubiquitination via two steps after activation of Ub molecule (Fig. 2A [59]). WWP1 exhibits the capability of directing both the mono and polyubiquitination of its target proteins. It can monoubiquitinate specific substrates including Gag [60], SPG20 [61] and p53. Furthermore, WWP1 can polyubiquitinate various targets including PTEN, p27, KLF5, etc., through K27, K48 and K63 linkages (Fig. 2A [62]). Further research is required to determine whether WWP1 can polyubiquitinate its targets via other linkages (K6, K11, K29, K33) and uncover the full spectrum of its ubiquitin linkage types (Fig. 2A).

HECT type E3 ligases possess a catalytic cysteine that can form a covalent isopeptide bond with ubiquitin [63]. C890, the catalytic subunit of human WWP1, is responsible for ubiquitin transfer from WWP1 to its substrate [54]. E2 enzyme forms a binding interaction with the N-terminal region of the HECT domain, which is connected to the catalytically active side of the C lobe through a flexible hinge loop, facilitating the transfer of Ub. Mutation in this hinge loop hinders the ubiquitination of WWP1, highlighting the importance of the structural plasticity and the catalytic activity of the HECT domain. To avoid self-degradation by autoubiquitination, WWP1 adopts the multi-lock autoinhibitory mechanism involving both intramolecular and intermolecular autoinhibition [64]. This autoinhibitory mechanism depends on the regulatory signals and conditions within the cells. The intramolecular autoinhibition is mediated by the 2,3- linker located between its WW2 and WW3 domains and PY motif present within its HECT domain. The linker region between the WW2 and WW3 domains synergistically interacts with the HECT domain and inhibits the catalytic activity of the HECT domain (Fig. 2B, [59, 65–67]). Mutation in the linker region and HECT domain disrupts the enzymatic activity of WWP1, leading to various diseases including cancer. Studies by Courivaud et al. suggest that point mutation at E798V can hyperactivate WWP1 and disrupt its autoinhibitory function in prostate cancer (Fig. 2C, [59, 64]). Some studies have shown that phosphorylation in both the linker region and the HECT domain may induce a fully active state of WWP1 [68, 69]. The intermolecular autoinhibition is mediated by the homodimerization of two WWP1 molecules to inhibit its enzymatic activity. The inhibition is achieved through the binding of the 2,3-linker of one WWP1 to the HECT domain of the other and vice versa (Fig. 2D, [64, 70]). Germline variants of WWP1, K740N and N745S, can release WWP1 from its native autoinhibited state, leading to its activation [71, 72]. Thus, mutations in the HECT domain can inhibit WWP1 dimerization (Fig. 2E). Activated WWP1 promotes ubiquitination of PY-containing substrate proteins, thereby regulating their stability, localization, and activity. Thus, the catalytic mechanisms and autoinhibitory regulation of WWP1, particularly through HECT domain mutations and germline variants, plays a crucial role in determining its activity and ability to modulate the localization and activity of PPXY-containing substrate proteins. Increased understanding of these processes will have important impacts for potential therapeutic applications.

## Physiological functions of WWP1

**Fig. 3:**
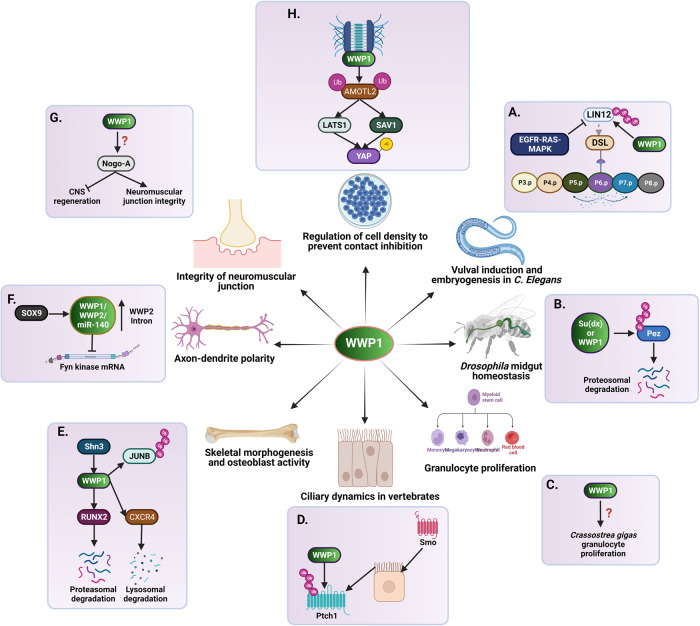
Diverse physiological functions of WWP1. **A** LIN-12 regulates the central vulval formation by interacting with DSL ligand. EGFR-RAS-MAPK signaling pathway inhibits LIN-12, whereas WWP1 promotes ubiquitination and degradation of LIN-12 to induce lateral vulva formation in *C. elegans*. **B** Suppressor of Deltex (Su(dx)), the *Drosophila* homolog of WWP1 ubiquitinates and degrades Pez protein to maintain *Drosophila* midgut homeostasis. **C** WWP1 regulates granulocyte proliferation in *Crassostrea gigas* through unknown mechanism. **D** WWP1 regulates ciliary dynamics in vertebrates by ubiquitinating Ptch1 and recruiting Smo. **E** Shn3 negatively regulates osteoblast function by recruiting WWP1 for the ubiquitination and degradation of RUNX2. Furthermore, WWP1 inhibits osteoblast differentiation and migration by proteasomal degradation of JUNB and lysosomal degradation of CXCR4. **F** SOX9 transcriptionally upregulates WWP1/WWP2/miR-140 and miR-140 represses *Fyn* kinase mRNA expression, which ultimately regulates axon-dendrite polarity. **G** WWP1 inhibits centra nervous system regeneration and maintains neuromuscular junction integrity by interacting with and regulating Nogo-A via unknown mechanism. **H** WWP1 maintains cell density by monoubiquitinating AMOTL2, which then interacts with LATS1 and SAV1 to phosphorylate YAP1.

WWP1 is a versatile protein expressed in almost all human tissues. It is localized in various subcellular compartments, including the plasma membrane (Sarcolemma), sarcoplasmic reticulum, mitochondria, endosome, and nucleus [43, 73]. WWP1 contributes to normal physiology through various biological processes, including its regulation of protein ubiquitination and interactions with several proteins (Fig. 3). WWP1(CeWWP1) plays a crucial role in *C. elegans* development by modulating morphogenesis during late embryogenesis [15]. LIN-12 acts as a key regulator of the patterning of vulval precursor cells (VPCs) during vulval induction in *C. elegans*. Activation of the EGFR-RAS-MAPK signaling cascade in the central VPC (P6.p) triggers the endocytosis and degradation of LIN-12. This relays a lateral signal to the neighboring VPCs (P5.p and P7.p) via activating DSL ligands. LIN-12 contains a di-leucine motif and a nearby Ser/Thr motif that mediate its internalization, whereas Lys residues are involved in its post-internalization trafficking. Studies by Shaye et al. have shown that WWP1 can promote LIN-12 degradation after its internalization and trafficking, a key function during vulval induction (Fig. 3A, [16]).

The protein tyrosine phosphatase Pez regulates intestinal stem cell proliferation and controls the Hippo pathway activity in *Drosophila* midgut epithelium. Wang et al. have shown that the *Drosophila* homolog of WWP1, Suppressor of Deltex (Su(dx)), can target Pez for degradation, thus promoting cell proliferation in the midgut epithelium and contributing to the maintenance of homeostasis in the *Drosophila* midgut (Fig. 3B, [74]). Interestingly, WWP1 plays an important role in the multiplication of the invertebrate oyster *Crassostrea gigas*, an economically important aquaculture species with a complex innate immune system that enables it to survive in harsh and constantly changing environments. Granulocytes are the primary immunocompetent hemoglobin-producing cells in oysters. Given their important role in the immune defence, their proliferation is strictly regulated by various signaling pathways to ensure haematopoiesis and a balanced immune response. Moreover, *Cg*WWP1, a WWP1 identified in oyster *Crassostrea gigas*, can regulate granulocyte proliferation in *Crassostrea gigas*, which may contribute to its survival (Fig. 3C, [75]).

In vertebrates, the Hedgehog pathway modulates ciliary dynamics by regulating the localization of Ptch1 and Smo. For instance, activation of the Hh pathway leads to the accumulation of Smo and its translocation into the cilia, which stimulates the evacuation of Ptch1 from the cilia, further promoting Smo activation and downstream signaling cascades. Lv et al. demonstrated that WWP1 can bind to Ptch1 and ubiquitinate Smo at basal levels, thereby regulating ciliary dynamics in vertebrates (Fig. 3D, [76]).

WWP1 can regulate osteoblast function, thus contributing to the maintenance of adult bone mass in humans. Schnuri-3 (Shn3) is a mammalian homolog of the *Drosophila* zinc finger adapter protein. It regulates adult bone formation and postnatal osteoblast activity and promotes the ubiquitination and proteasomal degradation of RUNX2, a transcription factor implicated in osteoblast differentiation (Fig. 3E, [17, 19, 20]). Mechanistically, physical interaction between Shn3 and RUNX2 decreases the protein stability and transcriptional activity of RUNX2 through a process involving the recruitment of WWP1, which targets RUNX2 for ubiquitination and proteasomal degradation. As expected, *Shn3* KO mice show increased RUNX2 protein levels and bone formation by osteoblast activity [17, 19, 20]. WWP1 inhibits the differentiation of mesenchymal stem cells (MSCs) into osteoblasts through ubiquitination and proteasomal degradation of JUNB and the lysosomal degradation of CXCR4 following induction by tumor necrosis factor (TNF), thereby promoting inflammation-mediated osteoporosis (Fig. 3E, [77, 78]). Interestingly, microRNAs (miRNAs) and siRNAs can negatively regulate WWP1 expression, thereby modulating bone regeneration and remodeling. For instance, miR-142-5p facilitates osteoblast function and matrix mineralization during bone healing by negatively regulating WWP1 expression levels in aged mice [79]. miR-15b promotes osteogenic differentiation in bone marrow mesenchymal stem cells (BMSCs) by inhibiting WWP1-mediated KLF2 degradation and inactivating the NF-kB signaling pathway [21]. These findings offer important insights into the molecular mechanisms that govern bone formation, suggesting novel targets for therapeutic strategies aimed at enhancing bone regeneration and repair. Understanding the interactions between WWP1 and miRNAs could lead to novel approaches in regenerative medicine.

WWP1 and WWP2 mediate the polarization of developing neurons through the SOX9/WWP1/WWP2/miR-140/Fyn regulatory pathway (Fig. 3F, [24]). Mechanistically, SOX9 transcriptionally upregulates *WWP1, WWP2*, and miR-140 (encoded by *WWP2* intron). miR-140, in turn, represses Fyn kinase mRNA, thus influencing axon-dendrite polarity acquisition and proper laminar distribution of developing neurons. Furthermore, Nogo-A, a member of the reticulon family, plays a dual role in the nervous system, inhibiting central nervous system regeneration while contributing to the maintenance of neuromuscular junction integrity. Qin et al. showed that WWP1 can interact with Nogo-A via the WW domain-PPPY motif (Fig. 3G, [23]), although the mechanisms by which WWP1 regulates Nogo-A remain unknown.

At higher cell density, WWP1 is localized and stabilized at cell junctions. This localization is mediated by its interactions with crumbs polarity proteins via the L/PPXY motif like Angiomotin-like 2 (AMOTL2). WWP1 directly binds and monoubiquitinates AMOTL2 at K347 and K408. Upon its monoubiquitination, AMOTL2 interacts with LATS2, facilitating the recruitment of SAV1. This, in turn promotes the activation of LATS2 kinase, which phosphorylates YAP and TAZ, resulting in their sequestration in the cytoplasm and subsequent degradation (Fig. 3H, [80]). This complex crosstalk between WWP1, LATS2, SAV1 and other members of the Hippo pathway highlights the role of posttranslational modifications in modulating the signaling cascades involved in cellular physiology and development. Increased understanding of these interactions can thus lead to the development of novel therapeutic strategies for various diseases. ERBB4, a member of the epidermal growth factor (EGF) receptor family, regulates the proliferation, survival, and differentiation of mammary epithelial cells. Research has shown that WWP1 can promote the ubiquitination and degradation of the CYT1 isoform of ERBB4, thereby inhibiting the proliferation of normal mammary epithelial cells [57]. Elucidating the role of WWP1 in CYT1 degradation underscores the potential for innovative treatment modalities targeting aberrant cellular proliferation in diseases such as cancer.

## Role of WWP1 in various diseases

**Fig. 4:**
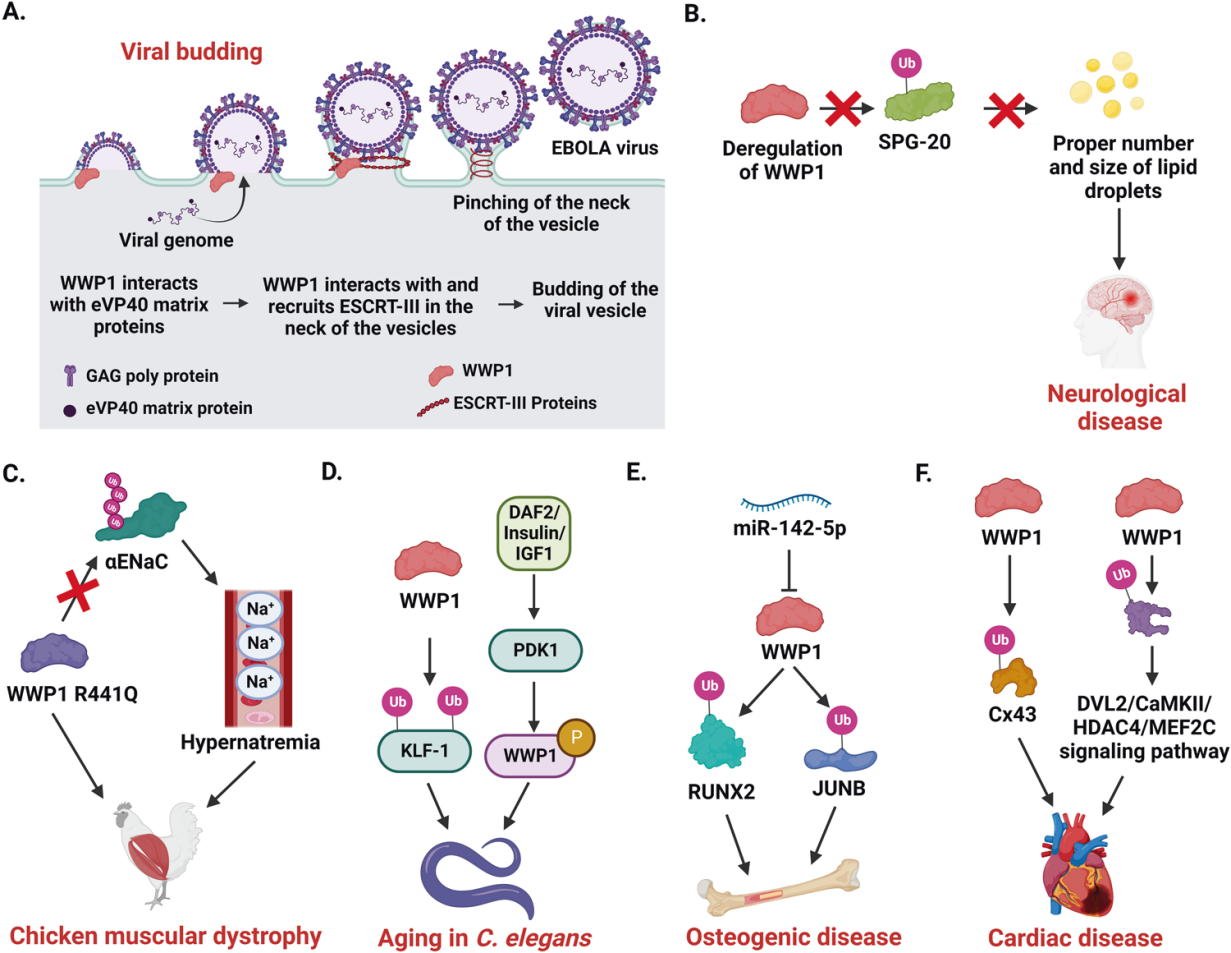
Role of WWP1 in various pathological conditions. **A** WWP1 plays crucial role in several viral diseases, where it helps in viral budding by interacting with host machineries. For instance, WWP1 interacts with and facilitates polyubiquitination of Ebola virus VP40 (eVP40) matrix proteins. Then, it interacts with and recruits endosomal sorting complexes required for transport III (ESCRT-III) complex to the neck of the viral vesicles, ultimately leading to the release/budding of the EBOLA virus particles. **B** WWP1 interacts with spastic paraplegia 20 (SPG20)/Spartin to promote its monoubiquitination, subcellular localization, and protein levels, thereby regulating the proper number and size of lipid droplets and ultimately maintaining proper neuronal health. Deregulation of WWP1 alters this pathway, leading to neurological diseases like Troyer syndrome. **C** WWP1 mutation (arginine to glutamine at 441, R441Q) impairs WWP1-mediated ubiquitination of αENaC (amiloride-sensitive epithelial sodium channel), leading to hypernatremia and chicken muscular dystrophy. **D** WWP1 regulates aging in *C. elegans* via two reported mechanisms. First, WWP1 directly interacts with and facilitates multiple monoubiquitination of KLF-1 (kruppel-like factor-1), an essential and specific regulator of dietary restriction (DR)-induced longevity in *C. elegans*. Second, WWP1 gets phosphorylated by the DAF-2 insulin/IGF-1 signaling pathway (crucial for aging in *C. elegans*) and therefore might be instrumental for aging. **E** WWP1 promotes osteogenic diseases such as osteoporosis via ubiquitination and degradation of RUNX2 and JUNB (crucial transcription factors for osteogenic differentiation), which is antagonized by miR-142-5p. **F** WWP1 promotes cardiac disorders such as left ventricular hypertrophy and lethal ventricular arrhythmias by ubiquitination-mediated degradation of Connexin 43 (Cx43). WWP1 also promotes pressure overload-induced cardiac hypertrophy via promoting K27-mediated polyubiquitination of disheveled segment polarity protein 2 (DVL2) and thereby enhancing the DVL2/CaMKII/HDAC4/MEF2C signaling pathway.

WWP1 plays a crucial role in normal physiology, and its deregulation can result in several pathological conditions. In the following sections, we will discuss the role of WWP1 in infectious diseases, neurological diseases, aging, and cancer (Table 1, Fig. 4).

**Table 1:** Role of WWP1 in Various diseases.

Disease type	Target	Role of WWP1	Reference
Infectious diseases (Ebola virus)	WW1 domain of WWP1 interacts with the Ebola virus eVP40 matrix (PPXY L-domain motif)	Reduces the cellular levels of eVLP40 oligomers and increases the assembly of virion and viral budding of VLPs.	[32]
Infectious diseases (Human T cell leukemia virus Type 1)	HTLV-1 Gag protein of PPPY and PTAP motif with amino terminal region of TSG101	Increases viral budding	[28]
Infectious diseases (prototypic foamy virus)	PFV Gag protein (PPXY motif)	Viral budding	[81]
Infectious diseases (Hepatitis B virus)	MLV Gag structural protein	Viral particle release	[31]
Infectious diseases (Murine Leukemia Virus)	MLV Gag of PPXY motif to the vacuolar protein sorting pathway	Viral budding	[8]
Infectious diseases (Prototypic foamy virus)	Arestin domain-containing protein 1	Viral budding	[82]
Infectious diseases (adenoviral disease)	Adenovirus Ad2 penton base protein at the N terminus (PPXY motif)	Virus internalization	[27]
Infectious diseases (COVID-19)	SARS-CoV-2 spike protein (25-PPAY-28)	Increase viral egression	[33]
Infectious disease (*Pseudomonas aeruginosa*)	DAF-2 insulin/IGF signaling pathway	Intrinsic cellular defence and innate immunity against PFTs	[83]
Normocephalic autism	Not determined	WWP1 Germline variants identified in ASD/ID/NDDs	[85]
Neurological diseases (Troyer Syndrome)	Spartin or SPG20	Reduce the level of SPG20 and prevents its accumulation on lipid droplets	[86]
Neurological disorder (Hereditary spastic paraplegias)	PPXY motif of Spartin	WWP1 acts as an adapter for SPG20 to recruit it to endosomes and lipid droplets	[61]
Neurological disorder	Intracellular domain III-IV linker region of the Ca_v_3.2 T-type channel	T-type calcium channels activity and nociceptive signaling	[87]
Neurodegenerative disorder (Dentatorubral and pallidoluysian atrophy)	Atrophin-1(5 PPXY motif) in the vicinity of the glutamine repeat	Promotes DRPLA disease	[4]
Chicken muscular dystrophy	WWP1 missense mutation (G1321A and R441Q)	Promotes muscular dystrophy	[89]
	R441Q missense mutation induces WWP1 degradation (autoinhibition)	Promote chicken muscular dystrophy	[43]
	Promote ubiquitination and degradation of β-dystroglycan	Promote dystroglycan related disorders	[92]
Hyperglycemia	KLF15	Increases skeletal muscle atrophy	[93]
Aging	DAF-2, DAF16, PDK1 and Insulin like growth factor-1	Modulates *C. elegans* lifespan and stress resistance.	[83]
	UBC18	Increases longevity and stress resistance	[94]
Cellular Senescence	p27^Kip1^	Inhibits the replicative senescence in fibroblast cells by degrading p27^Kip1^	[95]
Osteoporosis	RUNX2, JUNB and CXCR4	Decreases bone mass and bone formation in age-associated mice	[78]
	JUNB	Promotes inflammation-mediated osteoporosis and decreases differentiation into osteoblasts	[77]
Osteosclerosis	RUNX2	Regulates postnatal osteoblast activity by Shn3-mediated RUNX2 ubiquitination	[17]
Bone fracture healing	RUNX2	Elevates NF-kB members and promotes bone fracture healing	[97]
Type 2 diabetes	AMPKα2	In high glucose culture condition, WWP1 downregulates AMPKα2 expression on C2C12 cells	[22]
Obesity	Not determined	Regulates antioxidative reactions in adipocytes	[99]
	Not determined	Required for maintaining insulin sensitivity in obese white adipose tissue (WAT) and healthy mitochondrial function in obese WAT	[100]
Cardiomyocyte	Connexin 43	Left ventricular hypertrophy and the development of lethal ventricular arrhythmias	[101]
Atrial Fibrillation	Not determined	Promotes cardiac fibroblast proliferation	[105]
Hypertrophic heart	K27-linked polyubiquitination of DVL2	Promotes cardiac hypertrophy through the DVL2/CaMKII/HDAC4/MEF2C pathway	[102]
Cardiac atrophy	K27-linked polyubiquitination of DVL2	Regulate DVL2/CaMKII/HDAC4 axis-induced cardiac remodeling	[103]
Huntington’s	K63-mediated polyubiquitination of mHtt protein	Regulate mHtt protein levels, aggregate formation, and cell toxicity	[88]
Sepsis	K48-mediated polyubiquitination of NLRP3	YTHDF1 inhibit caspase 1 dependent pyroptosis by ubiquitinating to NLRP3, which leads to attenuation of sepsis	[84]

### Infectious diseases

Viruses and bacteria are responsible for various infectious diseases. Despite their diversity, viruses adopt a basic life cycle strategy when infecting the host cell. This strategy consists of various steps, including viral attachment to the host cell, entry, replication, and viral assembly and release. WWP1 might play a role in assisting the budding of both enveloped and non-enveloped viruses such as the VP40 (the Ebola virus (EBOV) VP40) matrix protein, which is involved in the virion assembly and budding of virus-like particles (VLPs). eVP40 interacts with WWP1 via its PPXY L-domain motif, which facilitates the efficient production of eVP40 VLPs during the budding process. WWP1-mediated ubiquitination of eVP40 increases the egress of VLPs, thus decreasing the cellular oligomerisation of eVP40 (Fig. 4A, [32]). Additionally, WWP1 can bind to the PPPYVEPTAP sequence located at the C-terminal MA region of HTLV-1 (Human T cell leukemia virus type -1) during viral budding [28]. WWP1 promotes viral budding in the prototypic foamy virus (PFV) via Gag ubiquitination [81]. HBV encodes PPAYRPPNAP, a late domain-like motif that recruits host WWP1, enabling the release of viral particles from infected cells [31]. Furthermore, WWP1 overexpression can result in the release of Hepatitis B Virus (HBV) from infected cells.[31]. Research also suggests that WWP1 can interact with and ubiquitinate the SARS-CoV-2 spike protein, which encodes a PPXY (25-PPAY-28 in spike protein) motif. Expression analysis has revealed that WWP1 and NEDD4 are overexpressed in COVID-19-infected patients. Rare germline variants of WWP1 and NEDD4 can promote viral egress and are associated with severe COVID-19 cases [33]. Mutations or deletion of the PPAY motif reduce the binding affinity of NEDD4 and WWP1, thus inhibiting viral budding.

The separation of cellular and viral membranes is a crucial step in the budding of enveloped viruses from infected cells. These viruses use the host multivesicular body (MVB) for this purpose. The execution of the MVB pathway, in turn, requires the endosomal sorting complexes for transport (ESCRT), composed of class- E vacuolar protein sorting (VPS) proteins. Serrano et al. reported that catalytically active WWP1 can induce viral budding by linking PPXY motifs to the host’s class-E VPS pathway [8]. Dominant negative (DN) WWP1 can inhibit the budding of murine leukemia virus (MLV), HTLV-1 and Ebola viruses [8, 28]. Similarly, WWP1-mediated ubiquitination of ESCRT-I drives PFV budding. WWP1 can also recruit ESCRT-III to regulate viral budding by interacting with arrestin domain-containing protein 1 (ARRDC1) [82].

WWP1 also contributes to the internalization of non-enveloped viruses. Penton base protein is an important component of non-enveloped viruses such as adenoviruses and is involved in the internalization of the virus and infection of host cells. Galinier et al., reported that WWP1 can interact with the PY motif of the penton base protein, suggesting that WWP1 might be implicated in virus internalization [27]. Nevertheless, it remains unclear whether this internalization is due to the WWP1- mediated ubiquitination of the adenovirus base protein or of its associated proteins. Nevertheless, these studies suggest that WWP1 holds promise as a potential target for therapeutic interventions aimed at countering viral infection.

Moreover, WWP1 is involved in bacterial pathogenesis. WWP1 might play a role in intrinsic cellular defence (INCED) and innate immunity against pore-forming toxins (PFTs) by regulating the DAF-2 insulin/IGF-1 signaling pathway. C. *C. elegans*, which has WWP1 is hypersensitive to pathogenic bacteria such as *Pseudomonas aeruginosa* and PFTs [83]. NLRP3 inflammasomes trigger inflammation and promote immune cell apoptosis, thus contributing to the worsening of sepsis progression. *WWP1* is a differentially expressed gene in sepsis recognized by YTH domain-containing family protein 1 (YTHDF1), an RNA-binding protein specialized in recognizing m6A, a dynamic mRNA modification that plays a pivotal role in governing protein expression across various post-transcriptional stages. WWP1 might also contribute to YTHDF1-mediated alleviation of sepsis by promoting NLRP3 ubiquitination [84]. Further investigation is required to elucidate the role of WWP1 in mammalian immunity.

### Neurological disorders

WWP1 plays a crucial role in neurological diseases (Fig. 4B). Novelli et al. reported that heterozygous germline mutations of WWP1 might contribute to the development and etiology of autism spectrum disorder (ASD) (5% of cases) [85]. WWP1 can regulate neurological disorders by interacting with or ubiquitinating several key proteins. Spastic paraplegia 20 (SPG20)/Spartin is mutated in Troyer syndrome, a neurological disorder characterized by distal amyotrophy. The protein is recruited to the endosomes and lipid droplets (LDs) and can regulate the size and number of LDs. LDs, on the other hand, are crucial for maintaining healthy neurons [61, 86]. WWP1 might regulate the function of SPG20 by promoting its monoubiquitination, subcellular localization, and protein levels, thereby regulating LD turnover. Hence, loss of WWP1-mediated regulation could result in the development of Troyer syndrome (Fig. 4B, [61, 86]). Understanding the interaction between WWP1 and SPG20 is essential for elucidating the mechanisms underlying lipid metabolism and could lead to the development of novel therapeutic approaches for metabolic disorders. Moreover, T-type calcium channels are involved in the transmission of nociceptive signals in the primary afferent pain pathway. Garcia-Caballero et al. showed that WWP1 can promote the ubiquitination of the T-type channel Cav3.2, thus deregulating USP5 and leading to neuropathic and inflammatory pain [87]. WWP1 could contribute to the development of Huntington’s disease (HD) by positively regulating mutant huntingtin protein (mHtt) levels, modulating aggregate formation, and inducing cell toxicity through K63-mediated polyubiquitination of mHtt [88]. However, the precise mechanisms underlying WWP1’s effects remain unknown. In conclusion, WWP1’s involvement in various neurological disorders, including autism spectrum disorders, Troyer syndrome, Huntington’s disease, and neuropathic pain, highlights its significance and offers promising avenues for developing novel therapeutic strategies.

### Chicken muscular dystrophy

Muscular dystrophy (MD) is a set of genetic disorders that cause progressive weakening and loss of muscular mass. Matsumoto et al. reported that WWP1 is responsible for MD in chickens with abnormal muscle (AM) [89]. They detected the presence of a missense R441Q mutation in WWP1 in chickens with MD, but not in other species (Fig. 4C, [89]). Godfrey et al. failed to identify such mutations in human dystroglycanopathy patients (*n* = 33), suggesting that WWP1 might not be involved in human dystroglycanopathy [90]. Furthermore, Imamura et al. showed that the R441Q mutation in WWP1 leads to increased degradation and loss of membrane (sarcolemma) WWP1 levels, which could contribute to the pathogenesis of chicken MD [43].

The mineralocorticoid hormone aldosterone is required for vertebrate ionic-salt and water balance. The levels of aldosterone, Na+ concentrations, and plasma osmolality are higher in MD chickens than in white leghorn (WL) chickens [91]. In contrast, the mRNA levels of *αENaC* (amiloride-sensitive epithelial sodium channel) are lower in MD chickens than in WL chickens. WWP1 mutations can impair the ubiquitination of αENaC, thereby affecting its protein expression. Prolonged overexpression of αENaC protein in the cell can lead to hypernatremia, characterized by high sodium concentrations in the blood, in MD chickens (Fig. 4C). Furthermore, dystroglycan is a ubiquitous membrane protein that plays an essential role in regulating muscle regeneration, and its dysregulation leads to muscular dystrophy. WWP1 can ubiquitinate and degrade β-dystroglycan and might thus be implicated in the development of muscular dystrophy [92]. Mutation of WWP1 at R441Q increases the ubiquitination of both β-dystroglycan and WWP1, relieving the autoinhibition of its ligase activity. The competitive binding of dystrophin and utrophin with WWP1 protects β-dystroglycan from WWP1-mediated degradation, thereby ensuring the integrity of the dystrophin-glycoprotein complex, which supports muscle fibers during contraction [92]. The interactions between these various proteins are therefore crucial for maintaining muscle integrity and function. Increased understanding of the impact of mutations affecting these proteins can thus inform the development of novel therapeutic strategies.

Hyperglycemia or high blood sugar is a major risk factor for diabetes. Research indicates that it increases muscle atrophy through the WWP1/KLF15 pathway. Hyperglycemia results in the downregulation of WWP1, thereby preventing WWP1-mediated degradation of KLF15 in skeletal muscle of old diabetic animals [93]. Altogether, both chicken MD- associated mutations in WWP1 and diabetes--associated downregulation of WWP1 could lead to skeletal muscle atrophy. Hence, the intricate relationship between hyperglycaemia, WWP1/KLF15 and diabetes-related WWP1 downregulation underscores the complex factors contributing to skeletal muscle atrophy, shedding light on potential mechanisms across various contexts.

### Aging

WWP1 mutations significantly reduce the lifespan of *C. elegans*. Chen et al. showed that WWP1 is a critical modulator of the DAF2 insulin/IGF-1 signaling network, which is crucial for aging in *C. elegans* [83]. Additionally, WWP1 directly interacts with and facilitates the monoubiquitination of KLF-1 (kruppel-like factor-1), an essential and specific regulator of dietary restriction (DR)-induced longevity in *C. elegans* (Fig. 4D, [94]). Cao et al. reported that WWP1 is highly expressed in young human fibroblasts compared to old senescent fibroblasts and facilitates the ubiquitination and degradation of the cell cycle-dependent kinase inhibitor p27-kip in human fibroblasts [95]. Thus, WWP1 is implicated in the dietary restriction-mediated lifespan of *C. elegans* (Fig. 4D), sparking in elucidating its roles and mechanisms of action in mammalian longevity.

### Bone and other diseases

Bone development and resorption are strictly controlled processes crucial for persistent remodeling and equilibrium. RUNX2 is a master regulator of osteogenesis and is involved in osteogenic and adipogenic differentiation from mesenchymal stem cells [96]. WWP1 plays a vital role in osteoporosis through ubiquitination and degradation of RUNX2 [17] and JUNB [77, 78] and is a potential target of miR-142-5p during bone fracture healing. Agomir-142-5p treatment induces higher levels of RUNX2 and JUNB proteins, while antagomir-142-5p treatment downregulates their levels (Fig. 4E, [79]). This suggests that miR-142-5p regulates the expression of JUNB and RUNX2 and might be a promising target for therapeutic interventions related to bone development disorders. TNF-α- mediated upregulation of WWP1 facilitates the proteasomal degradation of JUNB in mesenchymal stem cells. However, this process is entirely inhibited in mice that lack the *Itch* gene (*Itch* KO mice), indicating that ITCH, another E3 ubiquitin ligase, is required for this degradation process [97]. In high glucose culture conditions, WWP1 mediates the ubiquitination and degradation of the metabolic energy sensor AMPK in skeletal muscle C2C12 cells [22, 98], which could impact metabolism and energy regulation in these skeletal muscles. Further studies are required to elucidate the exact mechanisms by which increased glucose impacts WWP1 activity and the physiological effects of AMPK downregulation in this context.

WWP1 is upregulated in a p53-dependent manner in obese white adipose tissue (WAT). WWP1 overexpression can reduce ROS (reactive oxygen species) levels, while WWP1 knockdown results in increased ROS levels, thus suggesting a positive correlation between antioxidative proteins. Thus, WWP1 is an obesity-inducible E3 ligase that can protect against obesity-related stress in WAT [99]. Further research is required to confirm these findings. Additionally, increased oxidative stress markers are associated with lower AKT levels, plasma insulin levels, and unchanged glucose levels in obese WAT in *Wwp1* KO mice. Moreover, *Wwp1* KO mice exhibit lower citrate synthase activity (a mitochondrial enzyme implicated in the Krebs cycle), an important process in energy production. These mice also show improvements in both glucose and insulin tolerance tests, despite being obese. These findings highlight the potential role of WWP1 in the antioxidative response and mitochondrial function in WAT [100].

WWP1 overexpression in cardiomyocytes leads to a significant reduction (90%) of cardiac connexin 43 (Cx43) through ubiquitination-mediated degradation. This causes left ventricular hypertrophy and the development of lethal ventricular arrhythmias (Fig. 4F, [101]). Zhao et al. showed that WWP1 is significantly increased in patients with cardiac hypertrophy and in mice subjected to TAC. *Wwp1* KO can protect the heart from transverse aortic constriction (TAC)-induced hypertrophy. WWP1 promotes pressure overload-induced cardiac hypertrophy by inducing K27-mediated polyubiquitination of dishevelled segment polarity protein 2 (DVL2), thereby enhancing the DVL2/CaMKII/HDAC4/MEF2C signaling pathway (Fig. 4F, [102]). WWP1 promotes cardiac hypertrophy and remodeling in response to simulated microgravity [103], although the specific mechanisms by which WWP1 exerts its effect remain unknown. Understanding the role of WWP1 in these processes could lead to the development of novel therapeutic strategies to mitigate cardiac changes caused by prolonged exposure to microgravity. Additionally, *Wwp1* KO has a significant inhibitory effect on the development of left ventricular hypertrophy and the progression to HFpEF (heart failure with a preserved left ventricular LV ejection fraction), which might have important implications for the development of therapeutic approaches against these conditions [104]. miR-21 reduces cardiac β/SMAD2 signaling pathway and upregulating WWP1 expression. Interestingly, patients with atrial fibrosis had higher levels of miR-21 despite having lower levels of WWP1 [105]. Further research is required to elucidate the roles of miR-21 in WWP1 in the pathogenesis of atrial fibrosis.

## Role of WWP1 in cancer

**Fig. 5:**
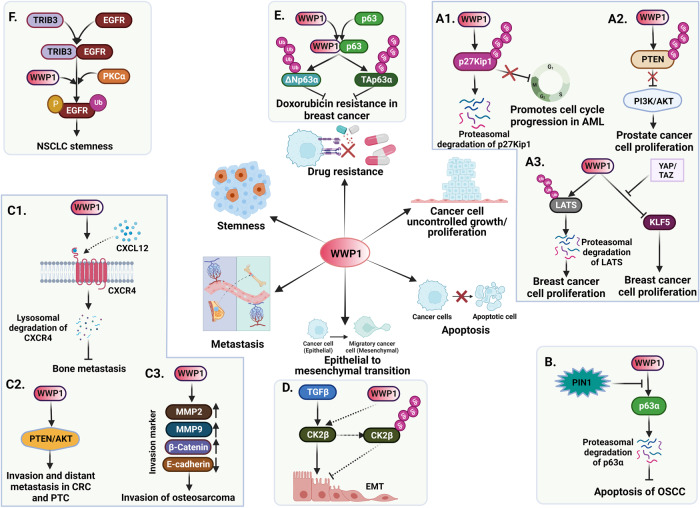
Functional implications of WWP1 in cancer. Schematic representation of the role of WWP1 in cancer. **A** WWP1 promotes growth and proliferation of cancer cells via different mechanisms. **A1** For example, WWP1 promotes polyubiquitination and proteasomal degradation of cell cycle inhibitor p27Kip1 and thereby facilitates cell cycle progression in AML. **A2** WWP1 promotes non-degradative K27-lnked polyubiquitination of PTEN, inhibits its dimerization and thereby activates PI3K/AKT signaling to promote cell proliferation in prostate cancer. **A3** WWP1 promotes breast cancer cell proliferation by facilitating proteasomal degradation of LATS1. In contrast, WWP1 plays a context-dependent tumor suppressor role in breast cancer by degrading oncogene KLF5, which is counteracted by oncogene YAP/TAZ. **B** WWP1 inhibits apoptosis in OSCC by enhancing proteasomal degradation of p63α, which is counteracted by PIN1. **C** WWP1 either promotes or inhibits cancer metastasis. **C1** WWP1 promotes CXCL12-mediated CXCR4 lysosomal degradation to inhibit bone metastasis. **C2** On the contrary, WWP1 promotes invasion and distant metastasis in CRC and PTC via enhancing the PI3K/AKT signaling. **C3** WWP1 also promotes osteosarcoma invasion by increasing the MMPs and β-catenin expression while decreasing that of E-cadherin **D** TGFβ enhances CK2β activation in a TGFβR-I kinase-dependent manner, and that activated CK2β promotes TGFβ-induced EMT. WWP1 leads to CK2β ubiquitination and proteasomal degradation to inhibit EMT. **E** WWP1 inhibits resistance to doxorubicin-induced apoptosis in breast cancer by promoting ubiquitination and proteasomal degradation of both ΔNp63 and TAp63. **F** WWP1 promotes the cancer stemness of NSCLC by promoting ubiquitination and stabilization of EGFR with the help of tribbles pseudokinase 3 (TRIB3).

Studies from the last two decades reveal that WWP1 is mutated, genetically amplified, and overexpressed in several human cancers, including solid tumors and hematological malignancies (Table 2, Fig. 5). WWP1 acts as a critical oncogene in many solid tumors, including breast, prostate, and hepatocellular cancers, and in hematological malignancies, specifically in AML. In other cancers, it acts as a tumor suppressor. WWP1 regulates cell growth, proliferation, apoptosis, EMT, migration, invasion, metastasis, drug resistance, and cancer stemness by targeting various substrates for polyubiquitination (Table 3, Fig. 5). The following sections explore the functions of WWP1 in cancer and their underlying mechanisms in detail.

**Table 2:** WWP1 expression and prognostic value in cancer.

Type of cancer	WWP1 expression (mRNA and/or protein)	*WWP1* amplification (Gene)	*WWP1* mutation (Gene)	Prognostic value	Model system	Refs
**Solid tumors**						
Breast cancer	Upregulated |	DNA copy number gain >2 fold	Dominant negative mutant WWP1C886S	ND	Cell lines	[122]
	Upregulated in 58% of cell lines	DNA copy number gain in 51% cell lines and 41% primary tumors.	ND	ND	Patient tissues, cell lines |	[41]
CRC	Upregulated	ND	ND	Poor prognosis (poor OS and DFS; larger tumor, higher distant metastasis and TNM stage)	Patient tissues, cell lines	[114]
CSCC	Upregulated	ND	ND	Poor prognosis (poor survival, higher histological grade, invasion rate and lymph node metastasis)	Patient tissues, cell lines	[117]
Gastric cancer	Upregulated	ND	ND	Poor prognosis (Median survival)	Patient tissues, cell lines	[110]
	Upregulated	ND	ND	ND	Patient tissues, cell lines	[139]
						[112]
Glioma	Downregulated	ND	ND	ND	Patient tissues, cell lines	[123]
HCC	Upregulated	ND	ND	Poor prognosis (poor OS and PFS)	Patient tissues, cell lines	[135]
ICC	Upregulated	ND	ND	Poor prognosis (Shorter OS, DFS and higher recurrence)	Patient tissues, mouse model, cell lines	[118]
Melanoma	Downregulated	ND	ND	Better prognosis (better OS)	Patient tissues, cell lines	[126]
NSCLC	Upregulated	ND	ND	Poor prognosis (poor OS)	Patient tissues	[146]
Oral cancer	Upregulated	Frequently amplified	Single base substitutions, A/G or C/T	ND	Patient tissues, cell lines	[111]
			transitions, and A/T or C/G transversions. |			
Osteosarcoma	Upregulated in 88% cancer tissues	ND	ND	ND	Patient tissues, cell lines	[115]
Prostate cancer	Upregulated	DNA copy number gain >2 fold	ND	ND	Cell lines	[122]
	Upregulated in 60% xenografts and cell lines	DNA copy number gain in 44% xenografts and cell lines and in 31% primary tumors	ND	ND	Patient tissues, cell lines, xenografts	[40]
	Upregulated	ND	ND	ND	Patient tissues	[150]
	Upregulated	ND	Point mutation at E798V	ND	Cell lines	[59]
**Haematological malignancy**						
AML	Upregulated	ND	ND	Poor prognosis (Poor OS)	Patient tissues, cell lines, NSG mouse model	[119]

**Table 3:** Diverse substrates and multifaceted role of WWP1 in Cancer.

Substrate	Effect on Stability	Mechanism of action	Role in cancer	Reference
PTEN	K27-linked nondegradative polyubiquitination	MYC-driven WWP1 activation inhibits PTEN dimerization and promotes AKT/PI3K signaling pathway	Suppresses PTEN dimerization, membrane recruitment, and promotes prostate cancer both in vivo and in vitro	[36]
	Polyubiquitination	Germline variants of WWP1 inhibits PTEN activation and hyperactivates PI3K signaling pathway	Promotes oligopolyposis and colon cancer	[71]
NDFIP1	Degradation	MYC-transactivated WWP1 promotes NDFIP1 degradation	Promotes proliferation, migration, invasion, and metastasis in ICC	[118]
TβR1	K63-mediated polyubiquitination and partially K48-mediated polyubiquitination and degradation	Point mutation of WWP1 hyperactivates WWP1 and degrades TβR1 in association with SMAD7	Promotes prostate cancer cell proliferation In vitro	[59]
	Degradation	Along with SMAD7, negatively affects the TGFβ signaling pathway	WWP1 inhibits transcriptional activity induced by TGF-β type-I receptor in human carcinoma cell line	[121]
CK2β	Degradation	CK2β required for TGF-β activation	Induces EMT in NSCLC	[141]
SMAD2, SMAD4 and TβR1	Degradation	Negatively regulates SMAD2, SMAD4 and TβR1 of TGF-β signaling pathway	Promotes cell proliferation in prostate cancer	[40]
KLF5	Degradation	KLF5 is protected by TAZ from WWP1-mediated degradation	Decreases breast cancer cell proliferation and tumorigenesis	[127]
	Degradation	KLF5 is protected by YAP from WWP1-mediated degradation and enhances FGFBP1 and ITGB2 expression	Decreases proliferation of human squamous cell carcinoma	[128]
	Degradation	ATXN3L deubiquitinates KLF5 and inhibits the expression of p27 and p21	Promotes breast cancer proliferation in vitro and tumorigenesis in vivo	[125]
	K48-linked polyubiquitination and degradation	BAP1 stabilizes KLF5 and activates PI3K-AKT-mTOR signaling pathway	Promotes melanoma progression and inhibits autophagy	[126]
	Degradation	αCatulin protects WWP1-mediated degradation of KLF5	Decreases EMT, and cancer stemness in NSCLC	[147]
	Degradation	Not determined	Promotes prostate Cancers	[122]
LATS1	Polyubiquitination, and Degradation	Interacts with the two PPXY motifs at 376 and 559 of LATS1	Promotes proliferation of breast cancer cells both in vitro and in vivo	[120]
TAp63α and ΔNp63α	Degradation	PIN1 increases p63α protein stability	Promotes proliferation and inhibits apoptosis in squamous cell carcinoma	[134]
p63 (ΔNp63α and TAp63α)	Degradation	Interacts with PPXY motif of p63α	Sensitizes breast epithelial cells to doxorubicin-induced apoptosis and cell survival by targeting p53 in breast cancer	[143]
ΔNp63α	Degradation	Metformin induces ΔNp63α protein instability by activating WWP1	Metformin induces cell detachment and cell death, Supresses growth and proliferation of human squamous cell carcinoma	[136]
EGFR	K63-mediated ubiquitination and stabilization	TRIB3 increases EGFR recycling and stability by recruiting PKCα to induce phosphorylation and ubiquitination at juxta membrane region	Promotes NSCLC resistance and stemness development in vitro and in vivo.	[146]
Estrogen receptor (ER)	Not determined	Positively regulates ER transcription	Promotes breast cancer cell growth	[106]
HER4 (JM-a/CYT1)/m80HER4	Membrane bound 80 kDa HER4 (m80HER4) degradation not full-length HER4	Not determined	Promotes growth and differentiation of breast cancer	[108]
ErbB4-CYT1	Degradation	Interacts with PPXY motif of ErbB4	Regulates biological activities of ErbB4 in breast cancer	[57]
CXCR4	Lysosomal degradation	CXCL12 increases bone metastasis by interacting with CXCR4 while WWP1 negatively regulates CXCL12 for CXCR4 lysosomal degradation	Decreases breast cancer bone metastasis by reducing cell migration and proliferation	[138]

### WWP1 in cell growth and proliferation

Cells in adult tissues exert stringent control mechanisms to regulate their cell cycle progression and maintain their cell number and organ size homeostasis. Cell cycle deregulation results in uncontrolled cell proliferation, which is one of the hallmarks of cancer. Studies suggest that WWP1 promotes cell growth and proliferation in various cancer types (Fig. 5A), including breast cancer [41, 106–108], prostate cancer [40], hepatocellular carcinoma (HCC) [109, 110], oral cancer [111], gastric cancer [110, 112], colorectal cancer (CRC) [113, 114], osteosarcoma [115], papillary thyroid carcinoma (PTC) [116], cutaneous squamous cell carcinoma (CSCC) [117], intrahepatic cholangiocarcinoma (ICC) [118] and AML [119].

RNAi-mediated WWP1 depletion results in cell cycle arrest at G0/G1 or G2/M phase in HCC, osteosarcoma, CSCC, and AML, suggesting that WWP1 can enhance cell proliferation by accelerating cell cycle progression [110, 115, 117, 119]. WWP1 promotes ubiquitination and degradation of the cell cycle inhibitor p27Kip1 in AML (Fig. 5A1) [119]. WWP1 also facilitates cell proliferation in ICC through ubiquitination and proteasomal degradation of NEDD4 family interacting protein 1 (NDFIP1) [118].

WWP1 regulates various signaling pathways, such as PI3K/AKT, TGF-β, and Hippo cascades, thus promoting cancer cell proliferation. WWP1 contributes to the growth and proliferation of gastric cancer, CRC, oral cancer, and PTC through the PI3K/AKT pathway [110, 111, 114, 116]. Lee et al. demonstrated that WWP1 can promote K27-mediated ubiquitination of the tumor suppressor PTEN, thus preventing its dimerization/oligomerization. This triggers its detachment from the plasma membrane, thus activating the PI3/AKT signal transduction pathway (Fig. 5A2) [36]. Interestingly, MYC can upregulate *WWP1* transcriptionally, leading to the activation of PI3K signaling and increased cell growth and proliferation [36, 40]. WWP1 gain of-function mutations were observed in two groups of individuals: those with PTEN hamartoma tumor syndrome (PHTS) and those with sporadic cancer [71, 72], suggesting that WWP1 can drive tumor growth.

The large tumor suppressor 1 (LATS1) is an S/T kinase and a tumor suppressor involved in Hippo signaling. WWP1 can negatively regulate and degrade LATS1 through the 26 s proteasome pathway, resulting in increased cell proliferation in breast cancer [120]. TGF-β signaling controls cellular responses leading to growth, survival, and differentiation using the heteromeric complexes of type-I and II transmembrane S/T kinases. Upon activation, these kinases phosphorylate specific intracellular signal transducers called SMADs (R-SMADs), including SMAD1, 2, 3, 5, 7, and 8. WWP1 can ubiquitinate TGF-β receptor-I (TβR-I) and SMAD4, leading to negative regulation of TGF-β signaling [40, 55, 112, 121], which promotes the proliferation of prostate and gastric cancer cells. Targeting these regulatory mechanisms might thus represent a strategy for modulating TGF-β signaling and improving treatment outcomes in these cancers.

Human kruppel-like factor 5 (KLF5) is an important transcription factor that regulates cell proliferation, differentiation, cell cycle regulation, and angiogenesis and suppresses cancer cell growth. WWP1 interacts with the PY2 motif of KLF5 (at codon 293-348) to promote its ubiquitination and degradation in breast and prostate cancers, suggesting that WWP1 might promote tumorigenesis via KLF5 downregulation (Fig. 5A3, [122]).

Despite its oncogenic role in promoting cell proliferation, WWP1 has been found to exert anti-proliferative effects in some cancers, thus highlighting its diverse and complex functions. For instance, WWP1 can suppress glioma proliferation by inhibiting the phosphorylation of the p65 subunit of NF-kB, thereby decreasing NF-kB-mediated miR-30a-5p transcription [123]. WWP1 interacts with the PY motif of the oncogene MUC1 to promote its ubiquitin-mediated lysosomal degradation, thus inhibiting cell proliferation and colony formation in breast cancer, liver cancer, and non-small cell lung cancer (NSCLC) [124]. Ge et al. have demonstrated that the deubiquitinase ATXN3L can bind to KLF5 to prevent its WWP1-mediated ubiquitination and degradation, which leads to breast cancer proliferation [125]. Hence, ATXN3L might be a potential therapeutic target for breast cancer. In melanoma, WWP1 mediates the K48-linked ubiquitination and degradation of KLF5, a process antagonized by oncogenic deubiquitinase BAP1 [126]. Increased understanding of these mechanisms can inform the development of novel therapeutic approaches. YAP and TAZ can also inhibit WWP1-mediated KLF5 degradation, leading to breast cancer proliferation (Fig. 5A3, [127–129]). These various studies indicate that WWP1 can act as a tumor suppressor depending on the context. The CYT1 isoform of HER4 (an epidermal growth factor receptor (EGFR) family member) inhibits cell growth and proliferation in breast cancer, suggesting that it could influence cancer sensitivity to Her-targeting treatment modalities. WWP1 targets HER4 CYT1 for ubiquitination and degradation, thereby preventing HER4 signaling and breast tumorigenesis [108], which could have implications for developing therapies aimed at modulating HER4 signaling in breast cancer.

### WWP1 in cell survival and evasion of apoptosis

Apoptosis plays a vital role in the development of multicellular organisms. Cancer cells can efficiently evade apoptosis, which represents a hallmark of cancer. WWP1 is frequently amplified in ERα (Estrogen receptor α)-positive breast cancer cells resistant to TRAIL (TNF-related apoptosis-inducing ligand)-induced apoptosis. Interestingly, TNBC cells are sensitive to TRAIL [41, 130–132], suggesting that WWP1 might contribute to drug resistance. WWP1 knockdown induces cell death in MCF7 and HCC1500 ER-positive breast cancer cells by activating the extrinsic apoptotic pathway, whereas WWP1 overexpression promotes cancer survival [133]. However, the exact mechanisms by which WWP1 is associated with TRAIL resistance but not with TNFα resistance in breast cancer remain unclear and require further research. Peptidyl-prolyl isomerase PIN1, which binds to phospho S/P or T/P residues, can regulate the TAp63 and ΔNp63 isoforms of p63. TAp63 contains a N-terminal transactivation domain (TAD) and functions as a tumor suppressor, whereas ΔNp63 lacks the N-TAD and acts as an oncogene. Mechanistically, PIN1 disturbs p63-WWP1 interactions in vitro and in vivo by binding to T538P, which is adjacent to the P550PXY543 motif. Hence, PIN1 prevents WWP1-dependent degradation of p63α proteins and ΔNp63-induced cell proliferation (Fig. 5B, [134]).

RNAi-mediated knockdown of WWP1 induces apoptosis in oral cancer [111], HCC [109, 135], breast cancer [133], osteosarcoma [115], gastric cancer [112], cutaneous squamous cell carcinoma cells [117] and AML [119]. WWP1 inhibits apoptosis by promoting cleaved caspase3 and p53 expression in HCC [109]. Furthermore, WWP1 prevents apoptosis by regulating BCL2 and BAX protein levels in osteosarcoma [115]. Metformin, an FDA-approved anti-diabetic drug, can promote cell death during glucose deprivation or glycolysis inhibition. Metformin exerts its effects through both AMP-dependent and independent mechanisms. For instance, metformin promotes ΔNp63α instability through AMPK- independent pathways, leading to disrupted cell-matrix adhesion and subsequent cell apoptosis. WWP1 plays a critical role in metformin-mediated inhibition of ΔNp63α stability. Metformin in combination with 2-DG (2-Deoxy-D-glucose) significantly inhibited xenograft tumor growth in vivo in human squamous cell carcinoma [136]. This combination thus warrants further investigation in the clinical setting to evaluate its efficacy.

### WWP1 in EMT, migration, invasion, and metastasis

Local invasion, migration, and distant metastasis enable cancer cells to spread into neighboring tissues, thus exacerbating cancer aggressiveness. RNAi and overexpression studies indicate that WWP1 facilitates migration and invasion in HCC [135], prostate cancer [137], breast cancer [138], CRC [113, 114], PTC [116], gastric cancer [139], LSCC [140], osteosarcoma [115], CSCC [117]. In breast cancer, WWP1 negatively regulates CXCL12-induced CXCR4 lysosomal degradation, thereby facilitating breast cancer cell migration and bone metastasis, leading to poor prognosis (Fig. 5C1, [138]). In CRC and PTC, WWP1 promotes invasion, metastasis, and TNM (Tumor; Node; Metastasis) stage by activating the PTEN/AKT signaling pathway (Fig. 5C2, [114, 116]). WWP1 also promotes osteosarcoma invasion by decreasing the expression of MMPs and β-Catenin and upregulating E-Cadherin (Fig. 5C3, [115]). Additionally, WWP1 enhances CSCC migration and invasion by reducing BCL2, Cyclin D1, pSTAT3, and Matrix Metalloproteinase-2 [117]. Hence, WWP1 might represent a promising therapeutic target for these cancers.

Some reports suggest that WWP1 can also act as a tumor suppressor by inhibiting migration and invasion in some cancers. Studies by Zhao et al. showed that WWP1 can inhibit migration and invasion in glioma by negatively regulating NF-kB phosphorylation [123]. Jia and colleagues found that BAP1 promotes melanoma migration and invasion by antagonizing WWP1-mediated KLF5 ubiquitination and degradation, suggesting that WWP1 plays a negative role in regulating invasion and migration in melanoma [126]. Kim et al. have shown that TGFβ enhances CK2 activation in a TGFβR-I kinase-dependent manner, thus promoting TGFβ-induced EMT. WWP1 elicits TGFβ-induced CK2β ubiquitination and proteasomal degradation, suggesting that WWP1 might have a negative role in EMT (Fig. 5D, [141]). Increased understanding of the dual roles of WWP1 can inform the development of personalized therapeutic approaches for cancer.

### WWP1 in drug resistance and cancer stemness

Drug resistance can lead to treatment failure and poor prognosis in many cancers. Zhou et al., have shown that WWP1 increases TRAIL resistance in breast cancer [133]. Wang and colleagues reported that WWP1 promotes resistance to paclitaxel (PTX) in TNBC via CircWAC/miR-142/WWP1 [142]. WWP1 binds to the PPXY motif of p63, disrupting both ΔNp63 and TAp63. Notably, inhibiting WWP1 enhances endogenous ΔNp63α levels in breast cancer, thereby conferring resistance to doxorubicin-induced apoptosis (Fig. 5E, [143]). As a result, targeting WWP1 may hold promise as a therapeutic approach for overcoming resistance to treatment in various cancer types.

Recent studies suggest that cancer stem cells (CSCs) drive metastasis and drug resistance [144, 145]. CSCs can self-renew and differentiate into various types of cancer cells. Research suggests that residual CSCs could mediate tumor relapse following conventional cancer therapy. WWP1 promotes cancer stemness in NSCLC by inducing ubiquitination and stabilization of EGFR (Fig. 5F, [146]). Tribbles Pseudokinase 3 (TRIB3), a stress sensor, interacts with EGFR, promoting its phosphorylation at T654 via PKCα and its ubiquitination at K689 through WWP1, thus leading to EGFR recycling, stability, and downstream activation of EGFR and NSCLC stemness. In contrast, Tung et al. demonstrated that the cytoskeletal linker protein, α-Catulin, can drive cancer stemness in lung cancer by interacting with KLF5 and antagonizing WWP1-mediated KLF5 degradation. These findings suggest that WWP1 could have a dual effect on cancer stemness depending on the context [147]. Elucidating the mechanisms by which WWP1 can promote or inhibit stemness will inform the development of novel therapeutic strategies for solid tumors.

## Transcriptional and posttranscriptional regulation of WWP1 in cancer

**Fig. 6:**
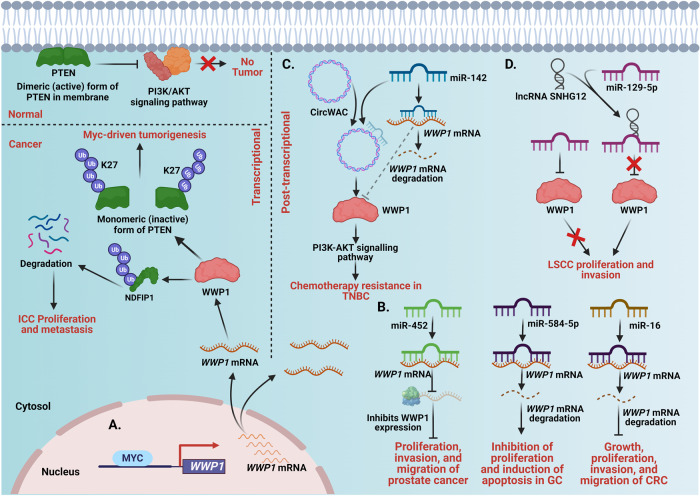
Regulatory mechanisms of WWP1 in cancer. WWP1 is regulated both at transcriptional and post-transcriptional levels in cancer. **A** In normal condition, the membrane dimeric form of PTEN inactivates the PI3K and AKT signaling pathway and thereby prevents tumor formation. However, the oncogenic transcription factor MYC transcriptionally upregulates WWP1 levels. WWP1 then promotes non-degradative K27 polyubiquitination of PTEN to inhibit its dimerization, membrane recruitment, and tumor-suppressive functions, leading to tumor initiation and progression. MYC-driven WWP1 upregulation also promotes NDFIP1 ubiquitination and degradation to promote proliferation and metastasis of ICC. **B** Post-transcriptionally, various tumor suppressor miRNAs such as miR-452, miR-584-5p, and miR-16 bind to 3′UTR of *WWP1* mRNA leading to its degradation or translation inhibition and ultimately attenuation of proliferation, invasion, migration of prostate cancer, gastric (GC) and colorectal cancer (CRC). **C** miR-142 (a tumor suppressor miRNA) binds with the 3’UTR of *WWP1* mRNA and degrades it. CircWAC, an oncogenic circular RNA, increases the expression of WWP1 by sponging miR-142 and activate PI3K-AKT signaling pathway and chemotherapy resistance in TNBC. **D** lncRNA SNHG12 sequesters the tumor suppressor miR-129-5p, upregulates WWP1 expression, and promotes the proliferation and invasion of lung squamous cell carcinoma.

The expression of WWP1 is tightly regulated by various transcriptional and posttranscriptional factors during cancer development (Fig. 6). For instance, MYC promotes the transcriptional upregulation of WWP1, which induces non-degradative K27 polyubiquitination of PTEN. This, in turn, inhibits its dimerization, membrane recruitment, and tumor-suppressive functions, leading to tumor initiation and progression (Fig. 6A, [36, 71]). Li et al. reported that MYC can directly bind to the *WWP1* promoter to upregulate its transcription, leading to NDFIP1 ubiquitination and degradation, which results in ICC proliferation and metastasis (Fig. 6A, [118]).

At the posttranscriptional level, the expression and function of WWP1 are regulated by various miRNAs (Fig. 6B, C, D). Goto et al. demonstrated that WWP1 is a target of miR-452, a tumor suppressor miRNA downregulated in prostate cancer. Mechanistically, miR-452 binds to the 3′-UTR of *WWP1* to inhibit its expression, thereby reducing WWP1-mediated proliferation, migration, and invasion of prostate cancer cells (Fig. 6B, [137]). miR-584-5p, which is downregulated in gastric cancer cells and tissues, targets the 3′-UTR of *WWP1* to promote its degradation and inhibit its proliferation, thus inducing apoptosis [112]. In gastric cancer, miR-129-5p targets two sites in *WWP1* CDS whereas miR-129-3p targets the 3′-UTR of *WWP1* to inhibit WWP1-mediated proliferation and migration in vitro and tumor growth in vivo [139]. Furthermore, miR-16 binds to the 3’UTR of *WWP1* and suppresses the growth, proliferation, invasion, and migration of colorectal cancer [113]. In contrast, oncogenic miR-30a-5p targets the 3’-UTR of *WWP1* to promote cell proliferation, migration, and invasion in glioma [123]. Studies suggest that oncogenic circular RNAs (circRNAs) and lncRNAs act as sponges for tumor-suppressive miRNAs, thus enhancing WWP1-mediated cancer progression. For instance, circWAC, which is highly overexpressed in TNBC and associated with poor prognosis, can act as a sponge for miR-142, thus promoting WWP1 upregulation, PI3K/AKT activation, and resistance to paclitaxel (PTX) in TNBC (Fig. 6C, [142]). Similarly, oncogenic lncRNA SNHG12 can sequester miR-129-5p to upregulate *WWP1*, thereby promoting WWP1-mediated proliferation and invasion of LSCC (Fig. 6D, [140]). Increased understanding of the mechanisms of action of these circRNAs can inform the development of novel therapeutic strategies for cancer.

## Therapeutic potential of WWP1 in cancer and other diseases

The multifaceted and complex roles of WWP1 in various cellular processes and disease pathways render it an attractive therapeutic candidate for cancer and other diseases. WWP1 plays an important role in viral budding, as previously mentioned. I3C, a natural small molecule inhibitor of WWP1, can inhibit viral egression and exert potent antiviral activity against viral infections such as COVID-19 [33] etc. The use of hydrogels containing siRNA against WWP1, complexed with nanoparticle (NP), at the site of murine mid-diaphyseal femur fractures, can enhance bone formation and mechanical strength [148] and represents a potential therapeutic approach for this condition. WWP1 expression is higher in the callus of fractured bones compared to non-fractured bones [97], suggesting that it may play a role in bone healing following fractures. C3A, a DNA aptamer, can bind to WWP1 to inhibit its ubiquitination ability, thereby increasing bone deposition for osteoporosis therapy [149]. These studies indicate that WWP1 might be a potential therapeutic target for bone fracture healing interventions. I3C also exhibits promising anticancer activities in preclinical models. For instance, treatment with I3C significantly suppressed tumorigenesis in MYC-driven and PTEN heterozygous mice by interacting with the WWP1 HECT domain, thus reactivating PTEN, and inactivating PI3K/AKT signaling [36, 71]. I3C, which is a natural compound produced by the breakdown of glucosinolate glucobrassicin, has emerged as a promising therapeutic approach for cancer due to its negligible toxicity.

Bortezomib, a proteasome inhibitor approved for multiple myeloma, can inhibit WWP1, preventing oncogenesis and bone metastasis in prostate cancer [150]. siRNA-mediated inhibition of WWP1 can significantly suppress tumor progression in many cancer types, such as breast cancer [41, 106–108], prostate cancer [40], HCC [109, 135], oral cancer [111], gastric cancer [110, 112], CRC [113, 114], osteosarcoma [115], PTC [116], CSCC [117], ICC [118] and AML [119]. Designing selective inhibitors that targets only cancer cells remains a major challenge in the field of drug discovery in cancer research. Most conventional therapeutics and inhibitors affect both normal as well as cancer cells, resulting in undesirable side effects and toxicity. Many inhibitors lose their efficacy over time due to the emergence of drug resistance, further limiting their therapeutic efficiency. Ongoing research is focused on identifying novel targets and developing selective and potent inhibitors capable of targeting specific proteins with minimal off-target effects. Several strategies can be leveraged to achieve increased selectivity and overcome resistance, including targeted therapy, combination therapy [151, 152], NP-based approaches, and immunotherapy.

Proteolysis Targeting Chimeras (PROTACs) are innovative heterobifunctional small molecules that offer promising avenues for cancer therapy. PROTACs use two binding sites to attach to a target protein and recruit an E3 ubiquitin ligase, triggering the degradation of the protein in question [153, 154]. One notable application of PROTACs is their ability to degrade the WWP1 oncoprotein, which has been implicated in WWP1-driven cancers. Moreover, these molecules can be specifically tailored to target oncogenic deubiquitinases (DUBs), such as ATXN3L and BAP1, which are known to destabilize the tumor-suppressive protein WWP1 in certain cancer types. Thus, PROTACs present a novel approach to selectively eliminate target proteins, including those that were previously considered undruggable. However, PROTACs come with certain limitations. These limitations encompass the complexity of their design, challenges in targeting transmembrane and aggregated proteins, the potential for drug resistance, issues with cellular permeability, oral bioavailability and concerns about toxicity [155–157]. Thus, the advancement of PROTAC depends on improving their stability, biodistribution, and cellular penetration. Such enhancements are crucial for their successful pharmacological application in cancer therapy [158].

DUBTACs (Deubiquitinase targeting chimeras), an extension of PROTACs technology, can recruit DUBs to counteract the degradation of tumor suppressors mediated by the WWP1 oncoprotein. This mechanism ultimately leads to tumor regression, offering a novel approach for cancer treatment. Similarly to PROTACS, DUBTACs may also encounter challenges and limitations [159]. Nevertheless, the promise of targeting WWP1-related conditions through these protein degradation approaches holds promise for the development of more efficient cancer therapies.

## Conclusion

In summary, WWP1 is a HECT type E3 ubiquitin ligase that interacts with PY motif-containing proteins, targeting them for ubiquitination and proteasomal degradation. WWP1-mediated ubiquitination also regulates protein localization and activity. WWP1 can modulate protein substrates without a PY motif through different adapters. Moreover, WWP1 is implicated in various physiological processes such as central nervous system regeneration, osteoblast differentiation, and *C. elegans* morphogenesis and embryogenesis (vulval development), through either ubiquitination or protein-protein interaction. Dysregulation of WWP1 expression and activity is associated with various pathophysiological conditions such as infectious diseases, neurological diseases, chicken muscular dystrophy, aging, osteogenic disorder, cardiac disorder, and cancer. In solid cancers, WWP1 plays a dual role as either an oncogene or tumor suppressor, depending on context and substrate interactions. This dual role thereby influences tumor growth, proliferation, invasion, migration, and EMT. Small molecule inhibitors, such as I3C, Bortezomib and siRNAs targeting WWP1, can effectively inhibit WWP1 expression and activity, thus exerting potent antiviral and anticancer activities. In conclusion, WWP1 emerges as a promising target for many diseases, including cancer.

## Future perspective

Recent studies have undoubtedly enhanced our knowledge of the roles of WWP1 in physiology and pathology. Increased understanding of the mechanisms underlying its functions and effects will elucidate its pathological roles and inform the development of novel therapeutic strategies targeting WWP1-related diseases. Although several studies indicate that WWP1 can act as both an oncogene and tumor suppressor, it is necessary to identify its substrates and targets in a cell or function-specific manner to understand the context-dependent underpinnings of its dual role.
